# Is infertility a disease and does it matter?

**DOI:** 10.1111/bioe.12495

**Published:** 2018-08-14

**Authors:** Hane Htut Maung

**Affiliations:** ^1^ Department of Philosophy School of Social Sciences University of Manchester Manchester United Kingdom

**Keywords:** assisted reproduction, disease, infertility, philosophy of medicine, state‐funded treatment

## Abstract

Claims about whether or not infertility is a disease are sometimes invoked to defend or criticize the provision of state‐funded treatment for infertility. In this paper, I suggest that this strategy is problematic. By exploring infertility through key approaches to disease in the philosophy of medicine, I show that there are deep theoretical disagreements regarding what subtypes of infertility qualify as diseases. Given that infertility’s disease status remains unclear, one cannot uncontroversially justify or undermine its claim to medical treatment by claiming that it is or is not a disease. Instead of focusing on disease status, a preferable strategy to approach the debate about state‐funded treatment is to explicitly address the specific ethical considerations raised by infertility. I show how this alternative strategy can be supported by a recent theoretical framework in the philosophy of medicine which avoids the problems associated with the concepts of health and disease.

## 1 INTRODUCTION

Infertility is characterized by the failure to conceive and is estimated to affect one in seven couples in the UK^1^ In the UK, state‐funded medical treatment for infertility is available under the National Health Service (NHS). Such treatment encompasses pharmacological interventions, surgical procedures, intrauterine insemination, and *in vitro* fertilization (IVF), with the latter two involving the use of either partner or donor gametes.^2^

The provision of state‐funded treatment for infertility is complemented by the fact that policy makers often consider infertility to be a disease. For example, according to the World Health Organization (WHO), infertility is “a disease of the reproductive system defined by the failure to achieve a clinical pregnancy after 12 months or more of regular unprotected sexual intercourse.”^3^ Similarly, in the *Report of the Committee of Inquiry into Human Fertilisation and Embryology*, infertility is considered to be a malfunction that warrants medical treatment:

On this analysis, an inability to have children is a malfunction and should be considered in exactly the same way as any other. Furthermore infertility may be the result of some disorder which in itself needs treatment for the benefit of the patient’s health. ... In summary, we conclude that infertility is a condition meriting treatment.^4^

This suggests that the disease status of infertility *is prima* facie assumed to provide a justification, or at least a minimal condition, for the provision of state‐funded treatment.

However, there is much disagreement in public discourse about the disease status of infertility. In 2002, the *British Medical Journal* ran an online survey to identify conditions considered to be “non‐diseases.” Infertility appeared in the list of contested conditions under the category “variant of normal.”^5^ A larger survey by Eli Adashi et al. of over 8,000 people demonstrated similar results, with only 38% of participants agreeing to the statement “infertility is a disease.”^6^ In academic discourse, too, there is disagreement about the disease status of infertility. Gay Becker and Robert Nachtigall argue that infertility is a social condition defined by the failure to meet cultural norms, which has been inappropriately medicalized and falsely recast as a disease.^7^ Similarly, Arthur Griel, Julia McQuillan and Kathleen Slauson‐Blevins argue that infertility is a socially constructed category shaped by pronatalism and patriarchy.^8^

The contested disease status of infertility is not only of theoretical interest to philosophers, but bears potential normative consequences for healthcare policy. As noted by Vardit Ravitsky and Raphaelle Dupras‐Leduc:

The implications of the question are clear: if perceived as a disease, public funding for its treatment is construed as justified and what remains to be determined is its prioritization in relation to other required treatments competing for limited resources … if not, funding it may not be justified from the outset.^9^

And so, given that disease status is often considered to be a justification or minimal condition for state‐funded treatment, it is tempting to think that a useful approach to evaluating whether or not infertility warrants state‐funded treatment is to establish whether or not infertility is a disease. Indeed, some philosophers have used this approach to defend the provision of certain kinds of treatment for infertility. For example, Arthur Caplan defends the provision of IVF,^10^ and Stephen Wilkinson and Nicola Williams defend public funding of uterus transplantation by arguing that infertility is a disease.^11^ Conversely, Emily McTernan argues that infertility fails to count as a disease in the relevant sense, and so does not have a claim to state‐funded treatment.^12^

However, in light of the contested disease status of infertility, others have suggested that invoking the disease status of infertility to justify or undermine its claim to state‐funded treatment is problematic. From a public health ethics perspective, Rebecca Brown et al. argue that focusing on the disease status of infertility is a distraction and instead propose that the discussion of state‐funded treatment should focus on the harms associated with involuntary childlessness.^13^ In this paper, I offer an analysis of the debate through the lens of philosophy of medicine. By exploring infertility through key approaches to disease in the philosophy of medicine, I aim to expose and unpack in greater detail some of the theoretical underpinnings of the controversy regarding infertility’s disease status. As we shall see, the trouble with infertility is that it is a highly heterogeneous category and different theories of disease turn out to disagree radically over what kinds of infertility qualify as diseases. I will also show how the ethical discussion of infertility’s claim to state‐funded treatment can be supported by a recent theoretical development in the philosophy of medicine, namely the antirealist approach to disease proposed by Marc Ereshefsky.^14^

The rest of the paper proceeds as follows. In the next section, I will present three leading philosophical theories of disease, namely Christopher Boorse’s bio‐statistical theory,^15^ Lennart Nordenfelt’s holistic theory of health,^16^ and Jerome Wakefield’s harmful dysfunction analysis.^17^ This will provide the theoretical backdrop against which to expose the problems regarding infertility’s disease status. In the third section, I will distinguish different kinds of infertility and evaluate each against the three aforementioned theories. The purpose is to reveal where there are disagreements between the theories with respect to what kinds of infertility qualify as diseases. In the fourth section, I will present Ereshefsky’s antirealist approach to disease and show how this could complement the proposal by Brown et al. to make the ethical considerations raised by the harms associated with infertility more explicit.

Before I proceed further, two clarifications are required. First, I do not aim in this paper to conclusively settle the debate of whether the aforementioned kinds of infertility ought to be treated using state‐funded resources. Rather, my aims are to understand the theoretical issues underlying the disagreement over infertility’s disease status and to show how an appropriate philosophical approach could support a more focused ethical debate about the provision of state‐funded treatment. Second, as with most philosophical work on disease, this paper uses the term “disease” in a broad sense as an umbrella concept that encompasses syndromes, injuries, disabilities and so on. Indeed, there are some contexts in which it is useful to distinguish these different sorts of condition. However, given that my philosophical analysis is concerned with the broad issue of whether infertility is a condition that falls within the remit of medicine, it suffices for the sake of my analysis to use “disease” in the aforementioned broad sense.

## 2 PHILOSOPHICAL THEORIES OF DISEASE

Philosophical theories of disease tend to fall into three broad categories. Naturalistic theories claim that whether something is a disease is purely a matter of biological fact.^18^ Normativistic theories claim that whether something is a disease depends on value judgements.^19^ Hybrid theories claim that both facts and values are required to determine disease status.^20^

I now explicate three particular theories of disease, namely the bio‐statistical theory, the holistic theory of health, and the harmful dysfunction analysis, which respectively exemplify naturalistic, normativistic and hybrid approaches. I have chosen to present these particular theories not necessarily because I endorse any of them, but because they represent some of the most influential examples of the aforementioned theoretical categories in the philosophical literature. Moreover, they capture many of those features which tend to be associated with conditions that are uncontroversially considered diseases. Hence, they are good examples to illustrate the spectrum of views on the concept of disease.

### 2.1 The bio‐statistical theory

Boorse puts forward a naturalistic theory of disease that defines it as a substandard deviation from normal biological function:

1. The reference class is a natural class of organisms of uniform functional design; specifically an age group of a sex of a species.
2. A normal function of a part or process within members of the reference class is a statistically typical contribution by it to their individual survival and reproduction.
3. Health in a member of the reference class is normal functional ability: the readiness of each internal part to perform all its normal functions on typical occasions with at least typical efficiency.
4. A disease is a type of internal state which impairs health, i.e., reduces one or more functional abilities below typical efficiency.^21^

Four features of Boorse’s theory are worthy of special note. First, Boorse assumes a teleological account of function, according to which the function of a part of a system is whatever it does that contributes towards achieving the system’s goals. In the case of a biological organism, Boorse takes the highest goals to be survival and reproduction. Hence, under this account, the function of the heart is to pump blood, because it is in virtue of its pumping blood that the heart contributes to the organism’s survival and reproduction. Second, an internal part can be a biological part, such as an organ or cell, or a psychological part, such as a mental module. Third, Boorse assumes a statistical account of normality, such that a part is functioning normally if it is contributing to the goals of a system with statistically typical efficiency, and abnormally if it is contributing below or above typical efficiency. Fourth, Boorse introduces the notion of a reference class, specifically an age group of a sex of a species, in order to limit the attribution of a function to sets of organisms smaller than the entire species. This is because what may be statistically typical for one set within a species may be atypical for another, such as the growth of bones being normal in children but abnormal in adults.

### 2.2 The holistic theory of health

In contrast to Boorse’s naturalistic theory, Nordenfelt puts forward a normativistic theory of disease, according to which the concepts of health and disease are determined by values:

A is completely healthy if, and only if, A has the ability, given standard circumstances, to reach all his or her vital goals. … A has a disease if, and only if, A has at least one organ which is involved in such a state or process as tends to reduce the health of A. The disease is identical with the state or process itself.^22^

Two features of Nordenfelt’s theory warrant special note. First, the vital goals of a person are characterized as “the set of goals which are necessary and jointly sufficient for his minimal happiness.”^23^ Hence, the theory explicitly depends on the value judgements of individuals. Second, Nordenfelt’s concept of disease requires the involvement of “at least one organ,” but such involvement need not constitute a deviation from statistically typical efficiency.

### 2.3 The harmful dysfunction analysis

Critical of both pure naturalistic and pure normativistic theories of disease, Wakefield develops a hybrid theory that integrates biological facts and value judgements. Note that Wakefield uses the term “disorder” rather than “disease”:

A condition is a disorder if and only if (a) the condition causes some harm or deprivation of benefit to the person as judged by the standards of the person’s culture (the value criterion), and (b) the condition results in the inability of some internal mechanism to perform its natural function, wherein natural function is an effect that is part of the evolutionary explanation of the existence and structure of the mechanism (the explanatory criterion).^24^

In contrast with Boorse’s teleological account of function, Wakefield endorses an aetiological account of function based on evolutionary theory, whereby “those mechanisms that happened to have effects on past organisms that contributed to the organisms” reproductive success over enough generations increased in frequency and hence were “naturally selected” and exist in today’s “organisms.”^25^ This is a popular account of function amongst philosophers of biology.^26^ Under this account, the function of the heart is to pump blood, because blood pumping is the mechanism of the heart that had causally contributed to the survival of an organism’s ancestors and to the evolutionary transmission of this mechanism to the present day organism. A dysfunction is a failure of this evolutionarily selected mechanism. According to Wakefield, such dysfunction is necessary but insufficient for something to be a disorder. In order to be a disorder, the dysfunction must also “cause significant harm to the person under present environmental circumstances and according to present cultural standards.”^27^ In contrast with Nordenfelt’s account which focuses on the person’s own assessment of his or her vital goals, the arbiter of harmfulness in Wakefield’s account need not be the individual bearer of the dysfunction. Rather, the dysfunction can be judged to be harmful according to social values. Nonetheless, it must be socially judged to be “direct harm to the individual, not just harm to society or other people.”^28^ Therefore, under the harmful dysfunction analysis, a biological fact about dysfunction and a social value judgement about harmfulness to the individual are jointly necessary and sufficient for something to be a disorder, or disease.

### 2.4 Discussion

Whilst extremely influential, these three philosophical theories of disease are not uncontested. Critics of the bio‐statistical theory have argued that its neglect of values does not reflect medical uses of health and disease.^29^ Moreover, if the claims about certain conditions being diseases are supposed to be value‐neutral, then it is unclear how they are supposed to justify normative judgements about whether we ought to treat these conditions.^30^ Other critics argue that the bio‐statistical theory fails to be genuinely naturalistic due to value judgements being implicitly invoked in its choices of goals, reference classes and measures of statistical typicality.^31^ A challenge that has been posed against the holistic theory of health is that it is too permissive, because it potentially medicalizes a wide range of afflictions that conventionally would not attract medical attention.^32^ The harmful dysfunction analysis aims to avoid such over‐permissiveness by setting both factual and evaluative constraints on the concept of disease, but it has still been argued that its evaluative component implies a counterintuitive relativism by suggesting that harmfulness is determined by the values of the particular society in which the bearer resides.^33^ The factual component has also been criticized for being of little clinical utility due to its explicit reliance on evolutionary theory, as well as for failing to be exclusively factual due to value judgements being implicitly invoked in its selective attributions of natural functions.^34^

Despite these criticisms, the three aforementioned theories of disease are very successful at capturing the disease statuses of many paradigmatic medical conditions. This is also in spite of the radical differences in their theoretical bases. For example, all three theories agree that myocardial infarction is a disease, albeit for different reasons. It is a disease under the bio‐statistical theory because it involves a part’s failure to perform its normal function with statistically typical efficiency for the relevant reference class; it is a disease under the holistic theory of health because an organ is involved in a state that compromises the ability of the person to achieve his or her vital goals; and it is a disease under the harmful dysfunction analysis because it involves a negatively evaluated failure of an evolutionarily selected biological mechanism. This can be said to apply to a wide range of paradigmatic medical conditions, including bronchial carcinoma, gastroenteritis, diabetes mellitus and so on. Therefore, given that they successfully capture the various features associated with uncontroversial disease states, the philosophical theories can serve as useful guides to our normative practices where they agree. However, as I shall show in the following section, infertility presents a controversial case where the theories disagree.

## 3 THE DAPPLED NATURE OF INFERTILITY

One of the challenges with determining the disease status of infertility is that infertility is not a unitary condition, but a heterogeneous category encompassing numerous states of affair. Given this heterogeneity, it is possible that some cases of infertility might be judged to be diseases, whilst other cases might not. In this section, I classify cases of infertility into four subcategories, which I call anatomical infertility, senescent infertility, relational infertility and social infertility. This classification is not intended to correspond to the ways in which cases of infertility are classified in medical theory and clinical practice. Rather, I have chosen this way of classifying cases of infertility because the resultant subcategories highlight issues that are of philosophical relevance to the problem of infertility’s disease status. I analyse each subcategory with reference to the aforementioned three theories of disease.

### 3.1 Anatomical infertility

This subcategory broadly refers to an inability to conceive that is for the most part attributable to a distinctive anatomical, physiological or genetic state or process located within the individual’s body. In men, this includes undescended testes, duct obstruction, varicocoele, surgical sterilization, endocrine insufficiency and chromosomal aneuploidy. In women, this includes congenital uterine anomaly, surgical hysterectomy, tubal obstruction, anovulation, pharmacological contraception, polycystic ovarian syndrome and chromosomal aneuploidy. Treatment approaches depend on the particular states or processes involved, but might include pharmacological therapies, surgical procedures and assisted reproductive technologies including use of donor gametes and IVF. In the UK, treatments for the aforementioned conditions are frequently provided under the NHS, although availability varies across different local commissioning groups.^35^

Under the bio‐statistical theory, anatomical infertility clearly qualifies as a disease. Each of the conditions mentioned above involves the failure of an internal biological part to perform its statistically typical contribution towards achieving the organism’s goal of reproduction at a statistically typical level of efficiency for the relevant reference class. This holds regardless of whether or not the individual evaluates his or her infertility as being undesirable. However, an implication of this theory is that people who are voluntarily using pharmacological contraception or who have had voluntary surgical contraception would be considered to have diseases.

According to the holistic theory of health, the disease status of anatomical infertility depends on the individual’s vital goals. Many cases of anatomical infertility are associated with distress because the bearers are unable to achieve their vital goals of having children.^36^ Nonetheless, there are also plausibly cases of people whose vital goals do not include procreation, for whom anatomical infertility would not be considered unwelcome. These include people who are using pharmacological contraception or who have had voluntary surgical contraception. Indeed, it has been argued that this dependence on people’s preferences is a reason not to consider infertility a disease.^37^ However, this is not unique to infertility, as there are many other conditions that are considered diseases despite only being associated with harm when the bearers have certain desires.^38^ For example, a mild limb injury may be welcomed by a pacifist if it confers exemption from military service, whilst a beef allergy may be welcomed by a vegetarian.^39^ The fact that some bearers of a certain condition do not find the condition undesirable does not preclude us considering the cases where the bearers do find the condition undesirable to be diseases. Hence, under the holistic theory of health, most cases of anatomical infertility qualify as diseases, but some do not.

The harmful dysfunction analysis also suggests that most cases of anatomical infertility qualify as diseases. All cases plausibly satisfy the criterion of biological dysfunction, as they involve failures of evolutionarily selected mechanisms. Moreover, such failures are generally deemed harmful or undesirable in a pronatalist society where parenthood is considered desirable.^40^ Given that Wakefield’s account considers the harmfulness judgement to be determined by social values rather than the individual bearer of the dysfunction, the fact that some bearers of anatomical infertility do not find the condition undesirable does not preclude its being considered a disorder in a pronatalist society. Indeed, Wakefield contends that “the ability to have children is commonly considered a benefit and its deprivation is commonly considered a disorder.”^41^ Again, exceptions might include cases of voluntary pharmacological or surgical contraception.

### 3.2 Senescent infertility

This refers to the decline in reproductive potential associated with advancing age. Such decline occurs in both sexes, but is more pronounced in women than in men. For that reason, the current discussion will focus on female senescent infertility. A study of 782 couples by David Dunson, Bernardo Colombo and Donna Baird found that the probabilities of pregnancy for women aged 19 to 26 were twice as high as those for women aged 35 to 39.^42^ A review by Cheryl Fitzgerald, Alison Zimon and Ervin Jones reports yearly pregnancy rates of 75% in women aged under 30, less than 55% in women aged over 40, and nearly zero in women aged over 45.^43^ According to the health website NHS Choices, around two thirds of women aged over 40 have fertility problems.^44^ In the UK, IVF is available under the NHS for women up to the age of 42.^45^ The Human Fertilisation and Embryology Authority report that a substantial proportion of women who received donor oocytes between 2009 and 2013 were aged over 45.^46^

Under the bio‐statistical theory, senescent infertility does not qualify as a disease, because what constitutes statistically typical function is established relative to an age group of a sex of a species. What is atypical earlier in life may be typical later in life. In light of the evidence that reproductive potential declines with advancing age, having a given capacity to conceive through unprotected heterosexual intercourse may be statistically typical for a woman aged between 25 and 30, but atypical for a woman aged between 40 and 45. Therefore, infertility in the latter case is not a disease under the bio‐statistical theory, because it is typical for the relevant reference class. What is less clear according to the theory, however, is at what age infertility goes from being abnormal to being normal. This is because the threshold between what is considered typical and what is considered atypical is not specified. Indeed, whilst Boorse states that functioning becomes abnormal when its efficiency falls some distance below the mean for the reference class, he concedes that “this distance can only be conventionally chosen, as in any application of statistical normality to a continuous distribution.”^47^ And so, the bio‐statistical theory suggests that infertility associated with advanced age is not a disease, but it does not tell us at what age being infertile becomes normal.

By contrast, under the holistic theory of health, senescent infertility can qualify as a disease if it impedes the bearer’s ability to achieve his or her vital goals. Again, this is dependent on whether or not the bearer’s vital goals include procreation. Of course, it is highly plausible that there are people of advancing age who do not wish to procreate, and so do not find senescent infertility undesirable. Nonetheless, the fact that a substantial number of women aged over 45 have been seeking assisted reproductive technologies in recent years suggests that many people with senescent infertility do find the condition distressing because it compromises their abilities to achieve their vital goals.^48^

Whether or not senescent infertility qualifies as a disease under the harmful dysfunction analysis is harder to determine. This is related to the fact that the harmful dysfunction analysis assumes an aetiological account of function based on evolutionary theory. It is contested amongst evolutionary biologists whether the decline in fertility with advancing age is a function, a non‐function, or a dysfunction. For example, it has been hypothesized that the cessation of fertility associated with menopause could be evolutionarily adaptive, because it allows a greater degree of maternal effort to be invested into enhancing the reproductive successes of living progeny.^49^ Others hypothesize that it is a sort of non‐functional epiphenomenon, such as a physiological trade‐off for efficient early‐life fertility^50^ or a by‐product of the relatively recent increase in human longevity.^51^ Such hypotheses, whilst plausible, are somewhat speculative and the evolutionary status of menopause remains inconclusive. Nonetheless, there is still a way in which senescent infertility could plausibly qualify as a disease under the harmful dysfunction analysis. According to an aetiological account of function, the function of the human ovary is to produce euploid ova, because such production of euploid ova is the mechanism of the ovary that had causally contributed to the reproduction of human ancestors and hence to the evolutionary transmission of this mechanism to the present day female human. The failure of the ovary to produce euploid ova that occurs with senescent infertility would therefore constitute a dysfunction. Furthermore, there is evidence suggesting that this decline in fertility associated with advancing age is negatively evaluated according to social values.^52^ Hence, senescent infertility would be considered a disorder according to this application of the harmful dysfunction analysis.

### 3.3 Relational infertility

This subcategory covers the scenario where a given couple are unable to conceive despite regular unprotected heterosexual intercourse, but each partner of the couple is typical with respect to his or her physiological capacity for reproduction. For example, each partner may have a reproductive capacity that lies within the statistical range considered normal, but towards the lower part of this range. The combined result is that the partners are unable to conceive with each other, but each partner could nonetheless conceive in various counterfactual situations with different partners.^53^ Relational infertility might also result from some sort of incompatibility between the partners that is peculiar to the specific couple. Again, each partner’s physiological capacity for reproduction is statistically typical, but they are unable to conceive with each other. Admittedly, the incompatibility hypothesis is speculative, but some researchers have suggested that it could be related to immunological blood group incompatibility.^54^

Relational infertility is philosophically interesting, because it suggests that the problem is not a property of any one individual, but of the couple as a whole. As noted by Melvin Taymor:

Individuals themselves are often neither ‘fertile’ nor ‘infertile’. … Here the significance of the couple as a unit plays an important role as to whether or not each individual will or will not be considered as having a ‘fertility problem’.^55^

Similarly, Allen Wilcox writes:

Two relatively infertile people may have difficulty conceiving with each other but be successful with other partners who are more fertile. Thus, an individual’s apparent fertility may change (for better or for worse) with a change in partners.^56^

This suggests a degree of externalism regarding infertility. That is to say, whether or not there is infertility does not solely depend on the internal state of the individual, but also depends to a large part on the state of affairs that extends beyond the individual, specifically the particular biological interactions between the partners of a couple. As we shall see, this has implications for what our three theories of disease say about the disease status of relational infertility.

Under the bio‐statistical theory, relational infertility would not qualify as a disease. Boorse defines health as “readiness of each internal part to perform all its normal functions on typical occasions with at least typical efficiency” and disease as “a type of internal state which impairs health.”^57^ The implication is that a disease is an internal state situated within the individual. As noted above, however, relational infertility is not situated within the individual, but is a property of the state of affairs that extends beyond the individual, namely the couple as a whole. Therefore, it is not a disease. A possible objection to this might be that relational infertility, whilst being dependent on the particular interpersonal context, still involves processes that occur within the individual. For example, the combined reproductive capacity of the couple still depends on the reproductive capacities of the individual partners and the couple’s immunological compatibility still depends on the immunological profiles of the individual partners. However, in reply to this, I concede that relational infertility does indeed rely on processes that occur within the individual, but such internal processes are statistically typical, and so are not diseased. The individual’s physiological capacity for reproduction is such that he or she could still conceive with typical efficiency in counterfactual situations with different partners, but happens to be in a specific situation where he or she cannot conceive.

The holistic theory of health, by contrast, allows for relational infertility to be a disease, as long as the person’s vital goals include having children with the particular partner. The theory suggests that a person has a disease if he or she “has at least one organ which is involved” in the state or process that impedes his or her ability to achieve his or her vital goals.^58^ Hence, an organ needs to be involved, but there is no requirement for this to constitute a deviation from statistical typicality. As noted above, whilst relational infertility involves the state of affairs external to the individual, this state of affairs still depends on facts about the reproductive capacities of the individual partners. These are facts that concern the states of organs. Therefore, it plausibly qualifies as a disease under the holistic theory of health.

The harmful dysfunction analysis yields a similar outcome to the bio‐statistical theory with respect to relational infertility. One of Wakefield’s criteria for disorder is “the inability of some internal mechanism to perform its natural function.”^59^ Again, the requirement for the mechanism to be internal to the individual suggests that relational infertility does not qualify as a disease because it is a property of a state of affairs that extends beyond the individual. Whilst internal processes are involved in relational infertility, they do not constitute failures of evolutionarily selected internal mechanisms, because each member of the couple still has the physiological capacity to conceive in counterfactual situations with different partners.

### 3.4 Social infertility

This subcategory broadly captures the absence of conception for reasons that are largely social or psychological rather than physiological. Examples include same‐sex couples and single persons by choice.^60^ Whilst social infertility does not strictly meet the WHO definition of infertility, it is nonetheless important to consider here because some cases are eligible for state‐funded treatment under current health policy. Presently in the UK, intrauterine insemination is available for women in same‐sex relationships, although it is recommended that NHS treatment should only be offered to women who have already failed to conceive after up to six cycles of privately funded donor insemination.^61^

Under the bio‐statistical theory, some cases of social infertility are not diseases, because they do not involve failures of internal parts to work at statistically typical levels of efficiency. These include people who cannot conceive because they have not found partners. Nonetheless, other cases could possibly qualify as diseases under the theory. For instance, the bio‐statistical theory suggests that homosexuality involves a disease of reproductive capability insofar as there is failure of a psychological module to contribute to reproduction at a statistically typical level of efficiency. This might seem untenable and philosophers have argued that such an unacceptable implication for the status of homosexuality indicates a serious problem with the bio‐statistical theory.^62^ Boorse himself is adamant that the bio‐statistical theory is supposed to be value‐neutral, and so a claim about whether there is failure of a part to contribute in a statistically typical manner to reproduction is intended to have no moral implications whatsoever.^63^ However, this supposed value‐neutrality highlights a limitation of the bio‐statistical theory when it comes to informing normative decisions about the provision and funding of healthcare resources.^64^ If a claim about a biological state is supposed to be value‐neutral, then it cannot tell us whether we ought to treat the biological state unless it is combined with an additional normative premise about health being valuable.

With respect to the holistic theory of health, social infertility clearly involves the inability to achieve one’s vital goals, and so qualifies as a state of impaired health. It could also be argued that there is sufficient organ involvement for it to qualify as a disease. In the case of a woman who is unable to conceive, there is organ involvement insofar as the uterus is not in the desired gravid state, regardless of whether the lack of male partner is because she is in a same‐sex relationship, single by choice, or unable to find a partner. However, it is less clear whether or how this analysis could be applied to men with social infertility.

Finally, under the harmful dysfunction analysis, many cases of social infertility are not disorders because they do not involve negatively evaluated failures of evolutionarily selected mechanisms. These cases include single persons who cannot conceive because they have not found partners. The theory is less clear with respect to people who cannot conceive due to being in same‐sex relationships. As Wakefield states, “the ability to have children is commonly considered a benefit and its deprivation is commonly considered a disorder, although even this has been disputed because of its implications for the classification of homosexuality.”^65^ Regarding the factual criterion of the theory, it is far from clear whether the development of homosexuality is adaptive or non‐adaptive from the perspective of evolutionary biology. Michael Ruse speculates that it may be evolutionarily adaptive, because it could allow more effort to be invested into enhancing the reproductive successes of genetically related kin.^66^ Regarding the evaluative criterion, Wakefield is clear that homosexuality is not a disorder because it is not harmful.^67^ However, as previously mentioned, the inability to conceive is viewed as being harmful in a pronatalist society. Therefore, whether or not the social infertility suffered by people in same‐sex relationships fulfils the harmfulness criterion depends on how the differing social attitudes towards homosexuality and childlessness are balanced.

### 3.5 Discussion

In this section, I have shown that different philosophical theories of disease disagree over which kinds of infertility do and do not qualify as diseases. The least controversial subcategory is anatomical infertility, most cases of which qualify as diseases under the three aforementioned theories. Nonetheless, there remain some cases of anatomical infertility over which the theories disagree. The other subcategories of senescent infertility, relational infertility and social infertility are more controversial, with equivocal outcomes and radical disagreements between theories. The findings are summarized in Table 1.

**Table 1:** Whether kinds of infertility can qualify as diseases according to different theories

	Bio‐statistical theory	Holistic theory of health	Harmful dysfunction analysis
Anatomical infertility	Yes	Yes	Yes
Senescent infertility	No	Yes	Yes
Relational infertility	No	Yes	No
Social infertility	Yes	Yes	No

The above analysis exposes some of the key theoretical sticking points that underpin the lack of consensus regarding infertility’s disease status. Infertility encompasses a diverse range of states, many of which do not possess all of the features that are commonly associated with disease. Some cases, such as cases of relational infertility and social infertility, do not involve failures of biological function. Other cases, such as cases of senescent infertility, may involve losses of biological function that are deemed undesirable, but these losses of biological function are statistically normal relative to the relevant reference class. Many cases are deemed harmful and stop the bearers from achieving their vital goals, even when they do not involve failures of biological function. Conversely, other cases may involve statistically atypical biological anomalies or failures of biological function, but may not be deemed harmful or stop the bearers from achieving their vital goals.

These findings are significant, because they suggest that we cannot legitimately invoke claims about infertility’s disease status to defend or criticize the provision of state‐funded treatment for infertility. As previously mentioned, the three theories of disease, despite their differences, successfully capture the disease statuses of many paradigmatic medical conditions, and so they can serve as useful guides to the normative practices of healthcare professionals where they agree. However, disagreements between the theories flag up controversial cases, such as the different subcategories of infertility, where consistent judgements about disease statuses are not available to inform such normative practices. And so, given the contested disease status of infertility, we cannot uncontroversially justify or undermine the provision of state‐funded treatment by claiming that infertility is or is not a disease.

## 4 REFRAMING THE DEBATE

The above controversy regarding infertility’s disease status indicates a need to reframe the debate about its claim to state‐funded treatment. Because infertility’s disease status is uncertain, it cannot be invoked to settle the debate. Moreover, given the ambiguities surrounding the concepts of health and disease, invoking such terms to settle the debate could distract from or obscure the specific considerations raised by the condition.^68^ Therefore, rather than invoking the contested concepts of health and disease, the debate needs to explicitly address the specific ethical considerations raised by infertility. This is the strategy endorsed by Brown et al., who argue that the ethical discussion about state‐funded treatment for infertility should explicitly consider the harms associated with involuntary childlessness, including suffering and the thwarting of valued life projects, as well as the social, cultural and economic factors that contribute to these harms.^69^

Whilst this strategy disputes the relevance of philosophical theories of disease to the debate about infertility’s claim to state‐funded treatment, I suggest that it can be complemented by a more recent theoretical development in the philosophy of medicine. In “Defining ‘health’ and ‘disease,’” Ereshefsky proposes an antirealist approach to disease that can encourage more explicit specifications of the ethical considerations relevant to these cases.^70^ Instead of using the concepts of health and disease, he suggests that medical discussions of controversial cases should be framed in terms of state descriptions and normative claims. A state description is a description of a physiological or psychological state. For example, the description a person’s red blood cells are rupturing is a state description.^71^ A normative claim is an explicit value judgement about whether the described state is desirable or undesirable. For example, the judgement that the rupturing of red blood cells is undesirable is a normative claim.

As an extension to Ereshefsky’s framework, I suggest that state descriptions need not be confined to descriptions of physiological and psychological states, but can also include details of social and environmental circumstances. This would allow the framework to more adequately capture cases of relational infertility and social infertility, but it also allows more comprehensive state descriptions of other conditions. For example, in addition to physiological and psychological details, a state description of a case of opioid dependence could include details about the person’s social environment, as these are also causally implicated in the condition. It is also worth noting that state descriptions do not employ explicit claims about normality, naturalness or function. This is because judgements about what is normal, natural or functional often carry evaluative or normative assumptions. To avoid invoking such evaluative assumptions as much as possible in the state descriptions, such notions are not overtly used. However, this is not to claim that state descriptions are entirely value‐neutral. Ereshefsky concedes that state descriptions may never be free from implicit values, because the scientific theories that inform them often implicitly assume value judgements.

The framework offered by Ereshefsky is intended to support discussions about controversial cases whose disease statuses are contested. When we cannot appeal to the disease status of a given condition, we can specify a description of the state of affairs and then examine whether the described state of affairs is considered desirable or undesirable. By distinguishing the state description from the normative claim, the framework helps us to locate more precisely where disputes may occur and how to resolve them. For example, in the debate about infertility, the disputes may be of two broad types. The first type of dispute is about what state of affairs is being discussed. As we have seen, infertility is a highly heterogeneous category, so there is room for misunderstanding with respect to what conditions are and are not being considered. The state description, then, exposes and helps to resolve any ambiguity or disagreement about the state of affairs on which the debate is focused. The second type of dispute is about whether or not the described state of affairs is undesirable. The evaluation of this requires explicit examination of the relevant harms and other ethical issues associated with the state of affairs.

And so, Ereshefsky’s theoretical framework supports the ethical strategy proposed by Brown et al. in three important ways. First and most obviously, it offers a way of framing the discussion about infertility that does not rely on establishing its disease status. Second, the state description specifies the physiological, psychological and social details of the kind of condition under discussion, which corrects for the ambiguity presented by the heterogeneous category of infertility. Third, the normative claim is informed by the explicit examination and evaluation of the suffering associated with involuntary childlessness, the thwarting of valued life projects, and the social, cultural and economic factors that contribute to these harms. The evaluation of these harms forms the ethical grounds for justifying the provision of state‐funded treatment for the condition.

Let us illustrate this through consideration of social infertility involving a woman in a same‐sex relationship who wishes to have a genetically related child. The state description would include biological facts about the woman’s reproductive physiology, as well as psychological and social facts about her sexual orientation and relationship status. The normative claim is a judgement about the extent to which this state of affairs is undesirable, which will be informed by the acute suffering associated with the inability to conceive and the more pervasive harm of being unable to form the desired family. It will also be informed by social, cultural and economic factors, such as the stigma associated with childlessness and the structural inequalities that make it difficult for people in same‐sex relationships to form their desired families. In turn, the consideration of these harms informs decisions about the provision of state‐funded assisted reproduction and population‐level interventions to address stigma, discrimination and structural inequalities.

Before I conclude, I entertain three potential objections. The first objection is that this approach does not distinguish problems that are considered medical from other sorts of problem, such as those considered social or moral, and so does not indicate why controversial cases should be managed by the healthcare sector rather than by a different sector. Ereshefsky addresses this concern by suggesting that the difference between a medical problem and another sort of problem is largely pragmatic, as it depends on which sector happens to have the expertise and resources to manage the problem.^72^ Due to various historical, cultural and scientific developments, it is the healthcare sector that has been afforded the expertise and resources for the management of infertility. Hence, the interventions for infertility tend to be provided by the healthcare sector. This is not to deny that social interventions are also important. Indeed, Brown et al. emphasize the importance of population‐level interventions to address the harms associated with involuntary childlessness, including sex education, public health programs and state facilitation of fostering and adoption.^73^ Furthermore, given that pronatalist social values are partly responsible for the suffering associated with infertility, there is a case for challenging these values at a social level through education and activism. Nonetheless, unlike the sorts of suffering associated with such oppressive social attitudes as racism, sexism and homophobia, it is plausible that the suffering associated with infertility is not solely attributable to pronatalist social attitudes. Some people might want children even if they did not live in a pronatalist society. Therefore, whilst social interventions to challenge pronatalism may be important, medical interventions for infertility may also turn out to be defensible options. By contrast, interventions such as skin whitening in a racist society or conversion therapy in a homophobic society would be indefensible, because the instances of suffering are solely attributable to the oppressive attitudes of the societies.^74^

The second objection is that it may still matter to the individual whether or not he or she is considered to have a disease, as this could influence society’s normative attitudes towards him or her. For example, it may matter to a person with opioid dependence that the condition is considered a disease because the disease label is perceived to legitimize the experience of suffering associated with the condition, whilst it may matter to a homosexual person that homosexuality is not considered a disease because the disease label is perceived to cast the person as abnormal and in need of medical treatment. Similarly, whether or not infertility is considered a disease may matter to a person with infertility because of such normative implications of the disease label. In response, whilst I concede that the disease label yields considerable normative influence, I argue that the conceptual and theoretical ambiguities concerning the label make it a blunt instrument. The label is not associated with a single kind of normative attitude, but with a diverse range of attitudes, including the perceptions that the bearer is not to blame, that the condition requires treatment, and that the bearer is abnormal. In a controversial case such as infertility, not all of these attitudes may be appropriate. Hence, invoking the disease label without further qualification could encourage unwanted attitudes whilst obscuring the considerations that inform these attitudes. An advantage of the approach I have endorsed is that it helps to clarify the ethical considerations raised by infertility, which enables us to be more explicit about the appropriate normative attitudes towards the condition.

The third objection is that state‐funded healthcare resources should be reserved for conditions that are unequivocally diseases, and so infertility’s claim to such resources is precluded by its contested disease status. In response, I suggest that the remit of medicine is not restricted solely to treating diseases, but also includes managing conditions not generally considered diseases. Examples include contraception to avoid pregnancy and analgesia in childbirth. Hence, whilst a condition’s being a bona fide disease can be taken to provide a *prima facie* justification for its claim to medical treatment, this in no way precludes equivocal cases from being evaluated with respect to their claims to healthcare resources.

## 5 CONCLUSION

Whether or not infertility is considered a disease is often assumed to provide a *prima facie* reason to treat or not treat it. However, different philosophical theories of disease disagree radically about what subtypes of infertility do and do not qualify as diseases. Given that the disease status of infertility is currently uncertain, we cannot uncontroversially justify or undermine the provision of state‐funded treatment for infertility by claiming that it is or is not a disease. Therefore, instead of relying on disease status, the debate needs to explicitly address the specific ethical considerations that are raised by infertility. This is a strategy that has been proposed by Brown et al. I have presented a philosophical framework, namely Ereshefksy’s framework of state descriptions and normative claims, which can facilitate such a strategy. This framework encourages us to make explicit the empirical details and value judgements that are relevant to discussions of infertility cases, hence making it possible to evaluate the provision of state‐funded treatment for infertility without necessarily invoking the concept of disease.

## CONFLICT OF INTEREST

The author declares no conflict of interest.

## Footnotes

1 Human Fertilisation and Embryology Authority (2013). *Fertility treatment in 2013: Trends and figures*. London: Human Fertilisation and Embryology Authority, p. 8.

2 National Health Service. (2017). *Treating infertility*. Retrieved May 20, 2017 from http://www.nhs.uk/Conditions/Infertility/Pages/Treatment.aspx

3 Zegers‐Hochschild, F., Adamson, G. D., de Mouzon, J., Ishihara, O., Mansour, R., Nygren, K., … Vanderpoel, S. (2009). International Committee for Monitoring Assisted Reproductive Technology (ICMART) and the World Health Organization (WHO) revised glossary of ART terminology, 2009. *Fertility and Sterility*, *92*, 1520–1524: 1522.

4 Department of Health and Social Security (1984). *Report of the Committee of Inquiry into Human Fertilisation and Embryology*. London, UK: Her Majesty’s Stationary Office, pp. 9–10.

5 Smith, R. (2002). In search of ‘non‐disease’. *British Medical Journal*, *324*, 883–885: 885.

6 Adashi, E. Y., Cohen, J., Hamberger, L., Jones, H. W. Jr, de Kretser, D. M., Lunenfeld, B., … van Steirteghem, A. (2000). Public perception on infertility and its treatment: An international survey. *Human Reproduction*, *15*, 330–334.

7 Becker, G., & Nachtigall, R. D. (1992). Eager for medicalisation: The social production of infertility as a disease. *Sociology of Health and Illness*, *14*, 456–471.

8 Greil, A. L., McQuillan, J., & Slauson‐Blevins, K. (2011). The social construction of infertility. *Philosophy Compass*, *5*, 736–746.

9 Ravitsky, V., & Dupras‐Leduc, R. (2014). Emerging legal and ethical issues in reproductive technologies. In Y. Joly & B. M. Knoppers (Eds.), *Routledge* *handbook of medical law and ethics* (pp. 223–243). London: Routledge, pp. 225–226.

10 Caplan, A. (1986). The ethics of in vitro fertilization. *Primary Care*, *13*, 241–253.

11 Wilkinson, S., & Williams, N. J. (2016). Should uterus transplants be state‐funded? *Journal of Medical Ethics*, *42*, 559–565.

12 McTernan, E. (2015). Should fertility treatment be state funded? *Journal of Applied Philosophy*, *32*, 227–240.

13 Brown, R. C. H., Rogers, W. A., Entwistle, V. A., & Bhattacharya, S. (2016). Reframing the debate around state responses to infertility: Considering the harms of subfertility and involuntary childlessness. *Public Health Ethics*, *9*, 290–330.

14 Ereshefsky, M. (2009). Defining ‘health’ and ‘disease’. *Studies in History and Philosophy of Biological and Biomedical Sciences*, *40*, 221–227.

15 Boorse, C. (1977). Health as a theoretical concept. *Philosophy of Science*, *44*, 542–573.

16 Nordenfelt, L. (2007). The concepts of health and illness revisited. *Medicine, Health Care, and Philosophy*, *10*, 5–10.

17 Wakefield, J. C. (1992). The concept of mental disorder: On the boundary between biological facts and social values. *American Psychologist*, *47*, 373–388.

18 Kendell, R. (1975). The concept of disease and its implications for psychiatry. *British Journal of Psychiatry*, *127*, 305–315; Boorse, *op. cit*. note 15.

19 Engelhardt, T. (1986). *The foundations of bioethics*. New York, NY: Oxford University Press; Nordenfelt, *op. cit.* note 16.

20 Reznek, L. (1987). *The nature of disease*. London, UK: Routledge; Wakefield, *op. cit.* note 17; Cooper, R. (2002). Disease. *Studies in History and Philosophy of Biological and Biomedical Sciences*, *33*, 263–282.

21 Boorse, *op. cit. *note 15, p. 555.

22 Nordenfelt, *op. cit.* note 16, p. 7.

23 Nordenfelt, L. (1995). *On the nature of health: An action‐theoretic approach* (2nd ed.). Dordrecht, Netherlands: Kluwer Academic Publishers, p. 90.

24 Wakefield, *op. cit.* note 17, p. 384.

25 Ibid: 383.

26 Millikan, R. G. (1984). *Language, thought, and other biological categories*. Cambridge, MA: MIT Press; Neander, K. (1991). Functions as selected effects: The conceptual analyst’s defense. *Philosophy of Science*, *58*, 168–184.

27 Wakefield, *op. cit.* note 17, pp. 383–384.

28 Wakefield, J. C. (2014). The biostatistical theory versus the harmful dysfunction analysis, Part 1: Is part‐dysfunction a sufficient condition for medical disorder? *Journal of Medicine and Philosophy*, *39*, 648–682: 664.

29 Reznek, *op. cit.* note 20.

30 Lewens, T., & McMillan, J. (2004). Defining disease. *The Lancet*, *363*, 664; Cooper, *op. cit.* note 20.

31 Cooper, *op. cit.* note 20; Kingma, E. (2007). What is it to be healthy? *Analysis*, *67*, 128–133.

32 Schramme, T. (2007). A qualified defence of a naturalist theory of health. *Medicine, Health Care, and Philosophy*, *10*, 11–17.

33 Nadelhoffer, T., & Sinnott‐Armstrong, W. (2013). Is psychopathy a mental disease? In: N. A. Vincent (Ed.), *Neuroscience and legal responsibility* (pp. 229–255). Oxford: Oxford University Press.

34 Ereshefsky, *op. cit.* note 14.

35 National Health Service, *op. cit.* note 2.

36 Monach, J. H. (1993). *Childless: No choice*. London: Routledge; Greil, A. L. (1997). Infertility and psychological distress: A critical review of the literature. *Social Science and Medicine*, *45*, 1679–1704; Cousineau, T., & Domar, A. (2007). Psychological impact of infertility. *Best Practice and Research: Clinical Obstetrics and Gynaecology*, *21*, 293–308.

37 Greil et al., *op. cit.* note 8.

38 Wilkinson & Williams, *op. cit.* note 11.

39 Schramme, T. (2007). The significance of the concept of disease for justice in health care. *Theoretical Medicine and Bioethics*, *28*, 121–135.

40 Miall, C. E. (1985). Perceptions of informal sanctioning and the stigma of involuntary childlessness. *Deviant Behavior*, *6*, 383–403; Kopper, B. A., & Smith, M. S. (2001). Knowledge and attitudes toward infertility and childless couples. *Journal of Applied Social Psychology*, *31*, 2275–2291; McQuillan, J., Stone, R. T., & Greil, A. L. (2007). Infertility and life satisfaction among women. *Journal of Family Issues*, *28*, 955–981.

41 Wakefield, *op. cit.* note 17, p. 384.

42 Dunson, D. B., Colombo, B., & Baird, D. D. (2002). Changes with age in the level and duration of fertility in the menstrual cycle. *Human Reproduction*, *17*, 1399–1403.

43 Fitzgerald, C., Zimon, A. E., & Jones, E. E. (1999). Aging and reproductive potential in women. *Yale Journal of Biology and Medicine*, *71*, 367–381.

44 National Health Service (2014). *Protect Your* *fertility*. Retrieved May 20, 2017 from http://www.nhs.uk/Livewell/Fertility/Pages/Protectyourfertility.aspx

45 National Health Service (2015). *Can I get IVF Treatment on the NHS?* Retrieved May 20, 2017 from http://www.nhs.uk/chq/Pages/889.aspx

46 Human Fertilisation and Embryology Authority, *op. cit.* note 1.

47 Boorse, *op. cit.* note 15, p. 559.

48 Human Fertilisation and Embryology Authority, *op. cit.* note 1.

49 Williams, G. C. (1957). Pleiotropy, natural selection, and the evolution of senescence. *Evolution*, *11*, 398–411.

50 Wood, J. W., O’Connor, K. A., & Holman, D. J. (1999). Biodemographic models of menopause. *Human Biology Association Abstracts*, *182*, 133.

51 Gosden, R. G., & Telfer, E. (1987). Numbers of follicles and oocytes in mammalian ovaries and their allometric relationships. *Journal of Zoology*, *211*, 169–175.

52 Harter, L. M., Kirby, E., Edwards, A., & McClanahan, A. (2005). Time, technology, and meritocracy: The disciplining of women’s bodies in narrative constructions of age‐related infertility. In P. Japp, L. Harter, & C. Beck (Eds.), *Narratives, health and healing: Communication theory, research and practice* (pp. 83–106). Mahwah, NJ: Lawrence Erlbaum Associates; Avila, B. E. (2016). *Importance of motherhood and/or social stigma of infertility: What’s driving infertility related outcomes?* (PhD Thesis) Michigan State University, East Lansing, MI. Retrieved May 25, 2017 from https://etd.lib.msu.edu/islandora/object/etd%3A3993

53 Taymor, M. L. (1990). *Infertility: A clinician’s guide to diagnosis and treatment*. New York, NY: Plenum Publishing Company; Wilcox, A. J. (2010). *Fertility and pregnancy: An epidemiologic perspective*. Oxford, UK: Oxford University Press.

54 Behrman, S. J., Buettner‐Janusch, J., Heglar, R., Gershowitz, H., & Tew, W. L. (1960). ABO (H) blood incompatibility as a cause of infertility: A new concept. *American Journal of Obstetrics and Gynecology*, *79*, 847–855; Schwimmer, W. B., Ustay, K. A., & Behrman, S. J. (1967). An evaluation of immunologic factors of infertility. *Fertility and Sterility*, *18*, 167–180.

55 Taymor, *op. cit.* note 53, p. 12.

56 Wilcox, *op. cit.* note 53, p. 69.

57 Boorse, *op. cit.* note 15, p. 555.

58 Nordenfelt, *op. cit.* note 16, p. 7.

59 Wakefield, *op. cit.* note 17, p. 384.

60 Appleby, J. B., Jennings, S., & Statham, H. (2012). Reproductive donation and justice for gay and lesbian couples. In M. Richards, G. Pennings, & J. B. Appleby (Eds.), *Reproductive donation: Practice, policy and bioethics* (pp. 211–230). Cambridge, UK: Cambridge University Press; Graham, S., & Braverman, A. (2012). ARTs and the single parent. In M. Richards, G. Pennings, & J. B. Appleby (Eds), *Reproductive donation: Practice, policy and bioethics* (pp. 189–210). Cambridge, UK: Cambridge University Press.

61 National Health Service (2017). Intrauterine insemination. May 31, 2017 from http://www.nhs.uk/conditions/Artificial-insemination/Pages/Introduction.aspx

62 Soble, A. (1987). Philosophy, medicine, and healthy sexuality. In E. E. Shelp (Ed.), *Sexuality and medicine, Volume I: Conceptual roots* (pp. 111–138). Dordrecht, Netherlands: D. Reidel Publishing Company; Cooper, *op. cit.* note 20; Kingma, *op. cit*. note 31.

63 Boorse, *op. cit.* note 15.

64 Lewens & McMillan, *op. cit*. note 30.

65 Wakefield, *op. cit*. note 17, p. 384.

66 Ruse, M. (1997). Defining disease: The question of sexual orientation. In J. M. Humber & R. F. Almeder (Eds.), *What* *is disease?* (pp. 135–172). Totowa, NJ: Humana Press.

67 Wakefield, *op. cit*. note 28.

68 Hesslow, G. (1993). Do we need a concept of disease? *Theoretical Medicine*, *14*, 1–14.

69 Brown et al., *op. cit.* note 13.

70 Ereshefsky, *op. cit.* note 14.

71 Ibid: 225.

72 Ibid.

73 Brown et al., *op. cit.* note 13.

74 Wilkinson & Williams, *op. cit.* note 11, pp. 561–562.

